# Optimization of cognitive assessment in Parkinsonisms by applying artificial intelligence to a comprehensive screening test

**DOI:** 10.1038/s41531-022-00304-z

**Published:** 2022-04-11

**Authors:** Paola Ortelli, Davide Ferrazzoli, Viviana Versace, Veronica Cian, Marianna Zarucchi, Anna Gusmeroli, Margherita Canesi, Giuseppe Frazzitta, Daniele Volpe, Lucia Ricciardi, Raffaele Nardone, Ingrid Ruffini, Leopold Saltuari, Luca Sebastianelli, Daniele Baranzini, Roberto Maestri

**Affiliations:** 1 Department of Neurorehabilitation, Hospital of Vipiteno (SABES-ASDAA), Vipiteno-Sterzing, Italy; 2 Department of Parkinson’s disease and Movement disorders Rehabilitation, Fresco Parkinson Center, “Moriggia-Pelascini” Hospital, Gravedona ed Uniti, Italy; 3 MIRT ParkProject, Livorno, Italy; 4 Fresco Parkinson Center, “Villa Margherita”, S. Stefano Riabilitazione, Arcugnano, Italy; 5 Neurosciences Research Centre, Molecular and Clinical Sciences Research Institute, St George’s University of London, London, UK; 6 MRC Brain Network Dynamics Unit, Nuffield Department of Clinical Neurosciences, Oxford, UK; 7 Department of Neurology, Franz Tappeiner Hospital (SABES-ASDAA), Merano-Meran, Italy; 8 Department of Neurology, Christian Doppler Medical Center, Paracelsus University Salzburg, Salzburg, Austria; 9 Department of Geriatrics, Memory Clinic, Franz Tappeiner Hospital (SABES-ASDAA), Merano-Meran, Italy; 10 Ergonomica SRLS, Varese, Italy; 11 Centre of Innovative Human Systems, Trinity College, Dublin, Ireland; 12 Department of Biomedical Engineering, Scientific Institute of Montescano - IRCCS, Istituti Clinici Scientifici Maugeri, Pavia, Italy

**Keywords:** Parkinson's disease, Pathology

## Abstract

The assessment of cognitive deficits is pivotal for diagnosis and management in patients with parkinsonisms. Low levels of correspondence are observed between evaluations assessed with screening cognitive tests in comparison with those assessed with in-depth neuropsychological batteries. A new tool, we named CoMDA (Cognition in Movement Disorders Assessment), was composed by merging Mini-Mental State Examination (MMSE), Montreal Cognitive Assessment (MoCA), and Frontal Assessment Battery (FAB). In total, 500 patients (400 with Parkinson’s disease, 41 with vascular parkinsonism, 31 with progressive supranuclear palsy, and 28 with multiple system atrophy) underwent CoMDA (level 1–L1) and in-depth neuropsychological battery (level 2–L2). Machine learning was developed to classify the CoMDA score and obtain an accurate prediction of the cognitive profile along three different classes: normal cognition (NC), mild cognitive impairment (MCI), and impaired cognition (IC). The classification accuracy of CoMDA, assessed by ROC analysis, was compared with MMSE, MoCA, and FAB. The area under the curve (AUC) of CoMDA was significantly higher than that of MMSE, MoCA and FAB (*p* < 0.0001, *p* = 0.028 and *p* = 0.0007, respectively). Among 15 different algorithmic methods, the Quadratic Discriminant Analysis algorithm (CoMDA-ML) showed higher overall-metrics performance levels in predictive performance. Considering L2 as a 3-level continuous feature, CoMDA-ML produces accurate and generalizable classifications: micro-average ROC curve, AUC = 0.81; and AUC = 0.85 for NC, 0.67 for MCI, and 0.83 for IC. CoMDA and COMDA-ML are reliable and time-sparing tools, accurate in classifying cognitive profile in parkinsonisms.

This study has been registered on ClinicalTrials.gov (NCT04858893).

## Introduction

Parkinson’s disease (PD) and atypical parkinsonian syndromes (APS) define the whole group of parkinsonisms, which are the major subsets of hypokinetic movement disorders. Nowadays, they represent a challenge for public health worldwide, because of their growing incidence in the population. The defective mesostriatal dopaminergic transmission in PD impairs both movement expression and action performing. The clinical spectrum includes motor symptoms (rigidity, resting tremor, bradykinesia, gait disturbances, postural abnormalities, and balance dysfunctions) and nonmotor symptoms. Nonmotor symptoms include sleep disorders, autonomic and gastrointestinal dysfunctions, sensory disturbances, motivational abnormalities, and cognitive deficits^1^. These latter may range from mild cognitive impairment (MCI) to impaired cognition (IC), as far dementia. Nonmotor symptoms can occur across all stages of PD and are key determinants of quality of life^1–3^. Progressive supranuclear palsy (PSP) and multiple system atrophy (MSA) are the most representative forms of neurodegenerative APS, whose etiology is different from that of PD. Vascular parkinsonism (VP) is a form of APS, whose features are of vascular origin, despite the symptoms are widely overlapped with those of other APS. As well as in PD, nonmotor features in APS determine a huge impact on quality of life and prognosis^4^.

The characterization of the right neuropsychological profile is pivotal to define the differential diagnosis with other neurological diseases and among different kinds of parkinsonisms^5–7^. Furthermore, it is also crucial to optimize both the pharmacological and rehabilitation treatments and, more generally, to improve the disease management^5,6^. For distinguishing between normal cognition (NC), MCI and IC in PD, the MDS-task force proposed two levels of evaluation: an abbreviated assessment (level I—L1) and/or a comprehensive assessment (level II—L2). The first one requires the administration of screening tests, while the second one requires large, in-depth neuropsychological batteries with at least two tests in each of the five principal cognitive domains: attention/working memory, executive function, language, memory, and visuospatial function^8^. To reach L1 classification, numerous screening tests have been proposed. Among them, the Mini-Mental State Examination (MMSE) and the Montreal Cognitive Assessment (MoCA) are widely adopted for evaluating global cognition^8–14^. Requiring no more than 10–15 min for administration, L1 evaluation provides an undoubtable advantage in terms of time-sparing in comparison with L2, which includes time-consuming neuropsychological tests. However, the two-level evaluation differs from at least two important aspects. First of all, by referring to the today-available cognitive screening tools, L1 provides clinicians with a classification along only two levels: scores higher than the cutoff indicate NC, while scores lower than the cutoff indicate MCI and IC, without distinction among the two classes^8^. Conversely, for L2 evaluation, MDS task force indicates the criteria for distinguishing NC from MCI and the last one from IC^15^. The second criticism refers to the low level of correspondence between L1 and L2 classifications^16^. In fact, several previous studies observed a discrepancy between the diagnoses obtained at L1 and those obtained at L2. Marras et al.^17^ found that the reliability of MoCA and MMSE is poor, when MDS task-force criteria were considered to define cognitive profile in PD patients. Moreover, comparing PD with parkinsonisms, Santangelo et al.^6^ observed very high percentage of impaired performances on L2 evaluation only in PSP patients, despite their MoCA score was within the normality range (mean score 20.1, when cut-off for Italian population is 15.5). Because frontal and executive functions are the most affected in PD and APS, FAB could be considered a valid alternative. In this concern, Eschlbock et al.^14^ studying cognition in MSA noted an impairment in executive functions in 40% of patients (evaluated with FAB), despite their MMSE was 27.6, making questionable the sensitivity of MMSE in detecting executive dysfunctions. Bezdicec et al.^18^ observed a specific relation between FAB scores and gray-matter density in frontal lobe of PD patients, recommending this test as a valid instrument for PD–MCI L1 screening.

Actually, while the weakness of MMSE and MoCA is the poor reliability, the weak point of FAB is to be too specific, thus losing the global vision in addressing patients’ cognition. In this study, we aim first at leveraging the screening capacity of MMSE, MoCA, and FAB by merging them into a single and composite measure, namely Cognition in Movement Disorders Assessment (CoMDA) (see Table 1). Further, we aim to overcome the limitations of L1 classification optimizing the screening-tool reliability of cognitive profile classification and reaching the 3-level class distribution. To reach these purposes, we referred to machine learning (ML). ML is a sub-area of artificial intelligence (AI) and excels in generating predictive models (both parametric and nonparametric) that learn effectively linear and nonlinear complex relationships among multivariate data patterns. ML has shown excellent accuracy and cross-generalization levels in diagnostic prediction^19,20^ and, therefore, such architectures are becoming increasingly important in the modern medical decision-making process^21^. The use of ML in healthcare has become common to carry out regression, classification, or unsupervised clustering tasks for various predictive models applied to a wide range of clinical uses^22^. Also, such predictive modeling capacity proved to outperform other more classical or deterministic solutions^22^. This is true, especially when the input variables (aka predictors) have nonlinear relations, abnormal distributions, or when they change over time. In fact, ML implementation is based on loss-function minimization methods, which allow to reduce the expected error in prediction. Another leveraging factor in referring ML models consists in their intrinsic capacity to improve the rate of accuracy and precision over time. Indeed, ML models improve their performances proportionally to resampling and retraining events. Such methods learn new patterns in the data and augment accuracy, precision, or sensitivity as their fine-tune parameters are exposed to learning based on new samples^23^.

**Table 1:** Items of MMSE, MoCA and FAB.

Cognitive domain	Test	Item	Score
Executive/Frontal functions	MoCA	1. Short version of Trail Making B task	0–1
	MoCA, FAB	2. Phonemic fluency task	0–1; 0–3
	MoCA, FAB	3. Verbal abstraction task	0–2; 0–3
	MoCA	4. Sustained Attention	0–1
	MoCA	5. Short Memory (backward digit span)	0–1
		6. Working Memory:	
		serial subtraction task;	0–3; 0–5
	MoCA	backward spelling task	0–3
	MMSE	7. Go/no Go task	
	FAB	8. Interference suppression	0–3
	FAB	9. Motor planning	0–3
	FAB	10. Clok´c-Drawing (visuo-spatial planning)	0–3
	FAB	11. Prehension behavior	0–3
			0–3
Visuo-spatial abilities	MoCA	1. Clok´c-Drawing	0–3
	MoCA	2. Copying of cube	0–1
	MMSE	3. Copying of two Pentagon	0–1
	MoCA	4. Short version of Trail Making B task	0–1
Memory	MMSE	1. Immediate recall	0–3
	MoCA	2. Delayed recall	0–5
	MMSE	3. Incidental recall	0–3
	MoCA	4. Short memory (forward digit span)	0–1
Orientation	MMSE, MoCA	1. Temporal Orientation	0–5; 0–4
	MMSE, MoCA	2. Spatial Orientation	0–5; 0–2
Language	MMSE, MoCA	1. Repetition	0–1; 0–2
	MMSE, MoCA	2. Naming (high frequency and low frequency)	0–2; 0–3
	MoCA, FAB	3. Fluency	0–1; 0–3
	MMSE	4. Oral Comprehension	0–3
	MMSE	5. Writing Comprehension	0–1
	MMSE	Writing Production	0–1

*MMSE* mini-mental state examination, *FAB* frontal assessment battery, *MoCA* montreal cognitive assessment.

Resuming, the aims of this study are (1) to define a new tool, named CoMDA, able to evaluate the global cognition with particular attention at frontal and executive functions; (2) to model and deploy ML algorithms, by implementing CoMDA-score as key predictor feature, to develop a powerful and fast screening tool with greater sensitivity in comparison with the available tools; and (3) to differentiate the patients’ performance along three classes of cognitive profile: “NC”, “MCI”, and “IC”.

## Results

### Baseline statistics and comparison substudy

The study population consisted in 400 PD, 41 VP, 31 PSP, and 28 MSA patients: Table 2 reports demographic variables of patients, grouped by disease.

**Table 2:** Demographic variables for all patients, grouped according to the disease.

Variable	All patients	PD	MSA	PSP	VP
Age	67.94 ± 9.26	67.48 ± 9.09	60.5 ± 8.77	70.97 ± 5.58	75.27 ± 8.19
Gender, n. and % of males	290 (58.0%)	239 (59.7%)	14 (50%)	19 (61.3%)	18 (54%)
Education (years)	10.63 ± 4.22	10.85 ± 4.19	10.61 ± 4.36	10.55 ± 4.06	8.54 ± 3.99
Disease Duration (years)	9.23 ± 5.33	9.99 ± 5.438	5.61 ± 2.51	6.03 ± 3.42	6.76 ± 4.08

*PD* Parkinson’s disease, *MSA* multiple system atrophy, *PSP* progressive supranuclear palsy, *VP* vascular parkinsonism.

The results of the method-comparison substudy are reported in e-Table 1 (Supplementary material) and confirmed that the CoMDA-derived MMSE, MoCA, and FAB scores are in excellent agreement with the values of the original source measures. The bias (i.e., systematic error) was nonsignificant for all scores and ranged from 1.2% (MoCA) to 0.4% (MMSE). According to the limits of agreement, one can be 95% confident that the error is less than 7% and 12% in the best case (MMSE) and in the worst case (FAB), respectively.

The CoMDA evaluation took 14.1 ± 1.1 min. This time was considerably longer than the time for assessing FAB and MMSE (4.1 ± 0.2 and 4.2 ± 0.4 min, respectively), but only about 44% more than MoCA (9.8 ± 0.6 minutes) and 28% less than the time needed for administering all three tests (18.0 ± 1.0 minutes).

### Statistics between disease groups

In e-Table 2 (Supplementary material), the percentage of normal performances to screening tests and in-depth neuropsychological tests according to normative data for Italian population are reported, stratified by disease.

Table 3 reports the results of nonparametric analysis of variance for screening and in-depth neuropsychological test scores for all groups of patients. Global group effect was significant for all screening tests (*p* = 0.032 for MMSE, *p* < 0.0001 for FAB, MoCA, and CoMDA). Post hoc analysis revealed only borderline significant differences in MMSE and CoMDA between PD and MSA, and in MoCA between PD and VP. At variance, largely significant differences in FAB, MoCA, and CoMDA were found between PD and PSP.

**Table 3:** Post hoc analysis of scores of screening tests and in-depth neuropsychological evaluations reported for all groups of patients.

Variable	PD	MSA	PSP	VP	*p*-value PD versus MSA	*p*-value PD versus PSP	*p*-value PD versus VP	*p*-value MSA versus PSP	*p*-value MSA versus VP	*p*-value PSP versus VP
MMSE	27.2 ± 2.3	26.2 ± 2.1	26.2 ± 3.5	26.8 ± 2.9	0.052	0.54	0.99	0.95	0.43	0.96
FAB	14.4 ± 2.7	13.8 ± 2.2	11.6 ± 3.9	13.5 ± 3.0	0.60	<0.0001	0.21	0.25	1.00	0.27
MoCA	23.4 ± 3.6	21.9 ± 3.7	20.5 ± 4.9	21.8 ± 3.7	0.13	0.003	0.057	0.97	1.00	0.92
CoMDA	65.1 ± 7.2	61.9 ± 6.4	58.1 ± 10.6	62.1 ± 8.2	0.060	0.0005	0.10	0.94	1.00	0.62
WCST	73.2 ± 36.7	68.9 ± 34.2	90.0 ± 22.1	77.5 ± 35.5						
Stroop test E	5.2 ± 7.6	3.2 ± 4.6	11.3 ± 8.4	11.3 ± 9.1	0.98	0.00031	<0.0001	0.003	0.001	1.00
Stroop test T	20.6 ± 13.0	25.6 ± 13.5	29.7 ± 26.4	22.8 ± 20.5						
TMT-A	44.3 ± 46.6	61.7 ± 35.6	70.0 ± 65.5	79.8 ± 88.7	0.016	0.17	0.010	0.99	1.00	1.00
TMT-B	126.3 ± 100.2	172.2 ± 79.8	161.6 ± 107.3	186.1 ± 123.6	0.014	0.42	0.024	0.96	1.00	1.00
TMT B-A	89.1 ± 81.8	112.3 ± 64.5	123.3 ± 87.1	132.1 ± 99.3	0.12	0.20	0.09	1.00	1.00	1.00
RAVLT-e	43.1 ± 10.2	44.6 ± 9.8	38.8 ± 8.3	40.9 ± 8.3	0.96	0.08	0.54	0.10	0.43	0.93
RAVLT-delayed recall	8.7 ± 3.2	9.3 ± 3.1	7.5 ± 2.9	7.8 ± 2.8	0.80	0.16	0.28	0.08	0.14	1.00
ROCF-c	29.3 ± 6.5	26.2 ± 6.1	19.3 ± 9.3	26.5 ± 8.1	0.022	<0.0001	0.11	0.28	0.95	0.014
ROCF-dr	14.9 ± 5.7	14.8 ± 6.3	11.8 ± 6.9	13.8 ± 4.5						
CF	43.5 ± 9.5	38.9 ± 9.7	30.9 ± 6.7	37.5 ± 8.6	0.18	<0.0001	0.003	0.023	1.00	0.044
PF	32.5 ± 10.5	25.2 ± 9.8	19.3 ± 7.6	25.8 ± 9.4	0.006	<0.0001	0.002	0.33	1.00	0.10

*PD* Parkinson’s disease, *MSA* multi system atrophy, *PSP* progressive supranuclear palsy, *VP* vascular parkinsonism, *MMSE* mini-mental state examination, *FAB* frontal assessment battery, *MoCA* montreal cognitive assessment, *CoMDA* cognitive screening in movement disorders assessment, *WCST* Wisconsin card sorting test, *Stroop Test E* stroop test errors score, *Stroop Test T* Stroop test time-in-seconds score, *TMT* trial making test, *RAVLT* Ray auditory verbal learning test, *ROCF* Rey–Osterrieth complex figure test, *CF* categorical fluency, *PF* phonemic fluency, *PD* Parkinson’s disease, *MSA* multi system atrophy, *PSP* progressive supranuclear palsy, *VP* vascular parkinsonism.

Regarding in-depth evaluation, global group effect was not statistically significant for WCST and ROCF-C (*p* = 0.10 and *p* = 0.17, respectively), while it was significant for all remaining tests. Post hoc analysis revealed several differences between groups of patients (see Table 3). On the other hand, as e-Table 2 describes, WCST and ROC-C stand out for being the most frequently impaired, in all groups, confirming that the executive and visuospatial ones are the most impaired cognitive functions in PD and APS.

### Predictive discrimination analysis

Cross-tabulations of L1 cognitive impairment by patients’ groups (‘1’ ≥ cutoff, ‘0’ = < cutoff) intercepted by MMSE, MoCA, and FAB are reported in e-Table 3 (Supplementary material), while L2 cognitive impairment cross-tabulation by patients’ groups is reported in e-Table 4 (Supplementary material). There is a highly significant association between L2 and disease-group distribution (Chi-Square *p* < 0.0001).

The comparison of MMSE, MoCA, FAB and CoMDA scores in NC and MCI + IC as assessed by L2 is reported in e-Table 5 (Supplementary material). It can be seen that MMSE, MoCA, FAB, and CoMDA scores are significantly worse in MCI and IC than NC as assessed by L2.

**Fig. 1:**
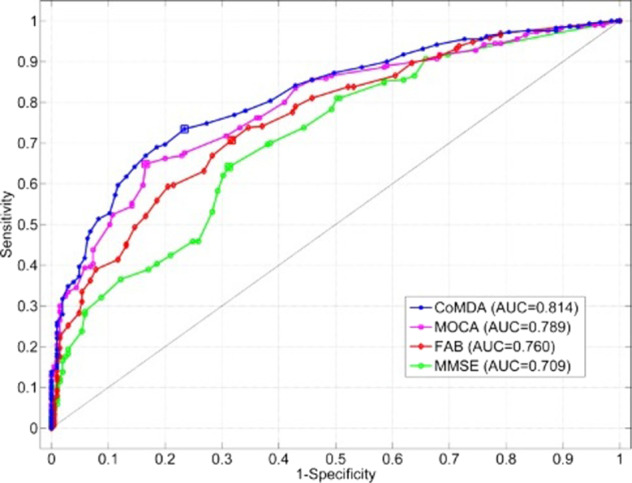
ROC curves. ROC curves obtained for the scores of the four cognitive-screening tools considered (see the text).

ROC analysis was used to quantify how accurately CoMDA, MMSE, MoCA, and FAB scores can discriminate between NC, MCI, and IC as assessed by L2. Figure 1 shows the ROC curves obtained for the four cognitive-screening tools. The AUC ranged from 0.708 for MMSE, to 0.789 for MoCA, to 0.759 for FAB, and 0.814 for CoMDA. Notably, contrast analysis for ROC curves revealed that AUC for CoMDA was significantly higher than those for MMSE, MoCA, and FAB (*p* < 0.0001, *p* = 0.028, and *p* = 0.0007) (see Fig. 1).

### Machine learning model: CoMDA-ML

A total of 15 different models were engineered and tested. They underwent a standard “10-fold cross validation technique” and e-table 6 reports the averaged metric performances for each estimator respectively. Cross-validation performance was based on a training set of 349 samples (~70% of total 500 patient-sample data) implementing a *k*-fold cross-validation procedure, *k* = 10. The trained algorithms were then scored on a final test/holdout set of 151 samples (remaining ~30% of sample data) to verify cross-generalization performance and the presence of undesirable model overfitting on unseen data (test/holdout set).

The predictive performance of the Quadratic Discriminant Analysis (QDA) algorithm showed higher overall-metrics performance levels across all algorithmic methods. Indeed, as shown in e-Table 6 (Supplementary material), the QDA algorithm resulted in the best-performing one across several competing estimators considered during the training validation process, outperforming other algorithms on the majority of the metrics.

This model maximized the bias-variance trade-offs, as it expresses optimal L2-prediction accuracy while maintaining appropriate cross-generalization performance.

Out of the 7 candidate predictors (see the statistics section—Machine learning), only 4 were selected by the QDA algorithm to predict 3-level L2 classification: “CoMDA score”, “age”, “disease duration”, and “years of education”. The resulting ML model was named “CoMDA-ML”.

The final model implemented a weighting on “CoMDA score” separately with “age” and “education”. These additional weightings defined two new predictor variables in the algorithm: “age*CoMDA score” and “education*CoMDA score”.

Among all candidate and new predictors, CoMDA score is the more informative one. In fact, as it can be seen in e-table 7 and e-table 8, the Information Gain tests revealed that CoMDA score provides the highest Information Gain, or the best-information entropy reduction, both in the original data sample and in the training set, where age*CoMDA and education*CoMDA features have been defined in the algorithm.

These variables, used to train the QDA, improved sensibly the predictive power. The final hyperparameters configurations of the finalized CoMDA-ML model are the exposed algorithm of the QDA: a classifier with a quadratic decision boundary, generated by fitting class-conditional densities to the data and using Bayes’ rule^24^.

QDA is derived by a target-class conditional distribution of the form

$$P(X|y = k),$$

and instantiation of Bayes’ rule for any given training sample

$$P(y = k|x) = \frac{{P(x|y = k)P(y = k)}}{{P(x)}} = \frac{{P(x|y = k)P(y = k)}}{{\mathop {\sum }\nolimits_l P(x|y = l) \cdot P(y = l)}},$$

where a targeted class *k* maximizes the posterior distribution with the log of the posterior

$$\begin{array}{ll}\log P\left( {y = k{{{\mathrm{|}}}}x} \right) = - \frac{1}{2}{{{\mathrm{log}}}}|{{\Sigma }}_k| - \frac{1}{2}(x - \mu _k)^t{{\Sigma }}_k^{ - 1}(x - \mu _k) + {{{\mathrm{log}}}}P(y = k)\\ + \, Cst,\end{array}$$

with $$Cst = P\left( x \right)$$.

(Source: Scikit Learn API, 2021—https://scikit-learn.org/stable/modules/lda_qda.html#lda-qda). The QDA predicted class is the one that maximizes this log-posterior (see also e-Table 9—Supplementary materials).

**Fig. 2:**
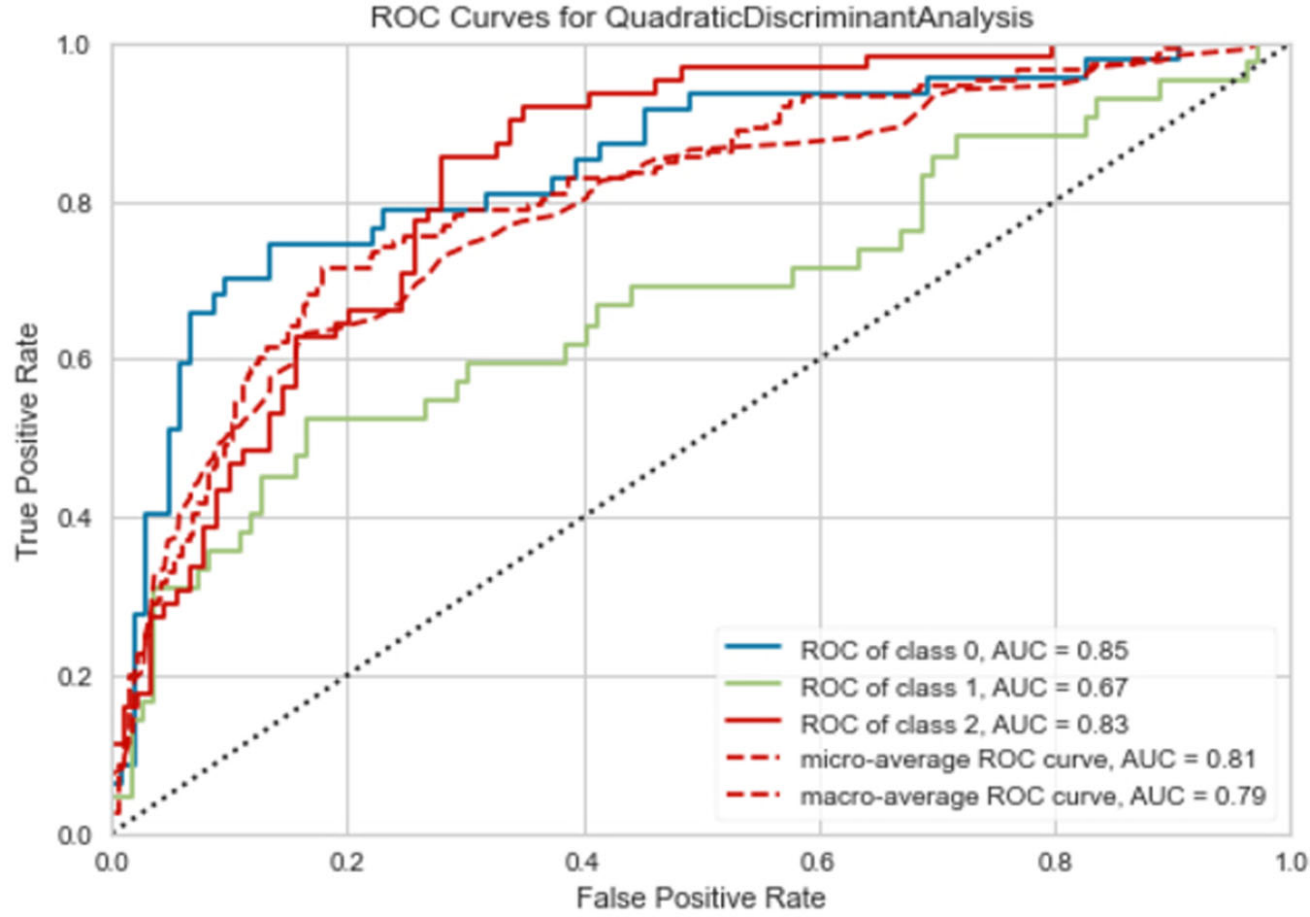
ROC curves for QDA. CoMDA-ML: ROC curves of multilevel classification.

The L2 multilevel-classification results are given by standard micro- and macro-average ROC curves, as well as individual class (one-vs-others) ROC curve values shown in Fig. 2. There is favorable convergence between micro- and macro-average AUC and ROC curve values at 0.81 and 0.79, respectively. Likewise, adequate single L2-class (one-vs-others) AUC ROC values have been achieved. Such findings replicate the ROC curve profiles obtained by the preceding baseline statistical tests. It is to note that micro-averaging combines all true/false-positive instances (across classes) to compute a single global ROC curve. Instead, macro-averaging pools together multiple ROC curve statistics as computed by each L2 class separately. Micro-averaging weights according to class dimension and macro-averaging equally weights across classes. Both are relevant to a complete multilevel assessment.

At the final test/holdout set CoMDA-ML resulted to provide accurately the 3-level multilevel classifications, having accuracy = 0.682, AUC = 0.81, sensitivity/recall = 0.682, precision = 0.681, F1 score = 0.676, Kappa = 0.508, and MCC = 0.513.

**Fig. 3:**
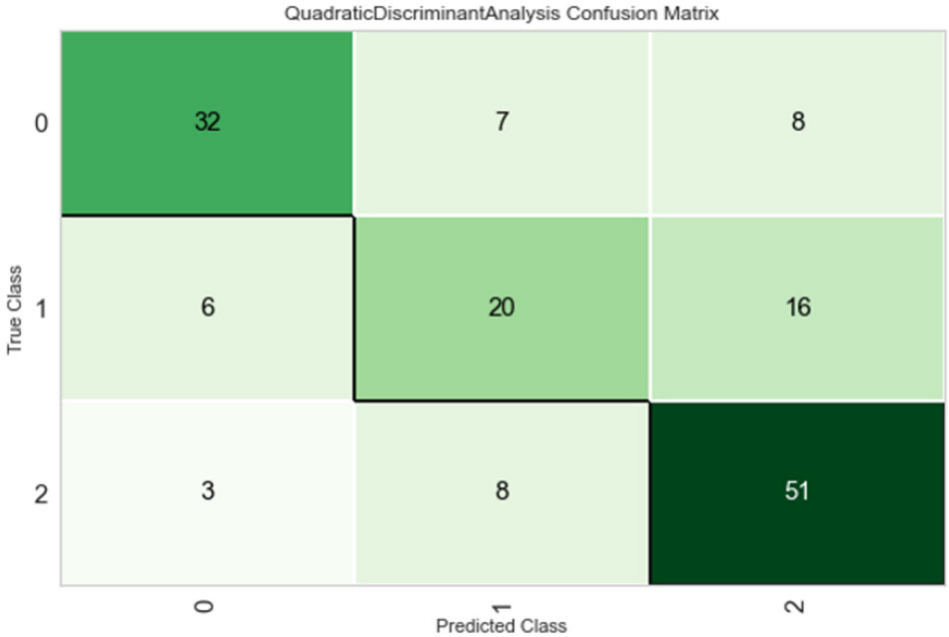
Confusion matrix. Accuracy-prediction levels for L2 classes as 0 = IC, 1 = MCI, and 2 = NC. Observed values (True Class) are reported in row-wise, while the predicted values (Predicted Class) are reported in column-wise.

The model confusion matrix at test/holdout set is given in Fig. 3 and it shows positive distribution rate of true positives in the main diagonal with respect to false-positive and false-negative rates.

Finally, e-Table 10 (Supplementary material) shows 10 random samples in the test/holdout set where the last two columns report the predicted L2 class (Label) besides the actual L2 class (L2): model-prediction results effective at this test-deployment outset: it can be seen that CoMDA-ML provides a reliable prediction of L2 classification.

## Discussion

The present study was aimed at creating CoMDA, a useful and time-sparing cognitive-screening tool thought to specifically evaluate the cognitive profile of PD and APS patients, and at developing CoMDA-ML to ameliorate the reliability of evaluation and to classify the patients’ performance along three classes of cognitive profile (“NC”, “MCI”, and “IC”), thus reducing the discrepancies between the classifications obtained with screening tests in comparison with in-depth neuropsychological batteries.

To the best of our knowledge, this is the first study in which AI is applied in cognitive-screening assessments to generate a prediction of the cognitive profile.

CoMDA has been built based on preexisting, frequently adopted screening tests, whose utilization is widely accepted, both in clinical and research settings. By merging the MMSE, MoCA, and FAB items, CoMDA guarantees a global evaluation, with particular attention on executive and frontal functions, specifically impaired in PD and APS patients. Furthermore, CoMDA-ML allows to improve the reliability of L1 evaluation and, finally, to classify the cognitive profile of PD and APS patients along three different classes: “NC”, “MCI”, and “IC”.

PD and APS share many signs and symptoms and present different rates of incidence in the population. These assumptions explain why our sample was not balanced among diseases, age, gender, and age at onset. As patients were continuously enrolled, the rate of PD and APS in our sample reflects the distribution of these pathological conditions in the general population^25^.

The results from the neuropsychological evaluation confirm the impairment of cognition in patients with PD and APS^26,27^. Fifty-nine percent of them get defective performances in one or more neuropsychological tests. We found that visuospatial deficits are the most common among PD and APS patients, followed by impairments in attention (specifically in interference-suppression capability), and executive functions, (mainly in mental flexibility). PSP patients manifest greater cognitive impairments than those suffering from other parkinsonisms. More than 50% of PSP patients showed pathological performances in WCST, TMT B–A, ROCF-c, ROCF-dr, and ST-E. Performances in ST-E and ROCF-c were defective in about 80% of PSP patients^6,28^. ROCF copy was the most impaired test among patients with MSA (67.9% of defective performances)^29^. The WCST test was defective in 42.7% of patients with PD^30^, thus confirming the executive dysfunction^31^. Therefore, deficits in executive functions remain the common neuropsychological denominator in all these pathological conditions. This finding may be prognostically valuable for identifying PD and APS patients at risk of dementia^7,27,32–34^. CoMDA is powerful in bringing together the intrinsic value of each single item belonging to the most used screening tests into a single instrument equipped with an effective elaboration processing, able to depict simultaneously both executive and visuospatial functions.

The results of the method-comparison substudy confirmed that the CoMDA-derived MMSE, MoCA, and FAB scores are in excellent agreement with the values of the original source measures. This is a point of strength of CoMDA, which can automatically provide the score of three well-known and widely adopted tests. By referring at nowadays-available normative data for these scores, we can observe that MMSE and FAB differ between patients and healthy controls and among the disease groups. Conversely, MoCA score did not differ between patients and healthy controls. This finding could be related to the low MoCA cutoff score identified in the normative data for the Italian population^35^.

MMSE, MoCA, and FAB scores showed a strong relation with L2, which is considered as the gold standard to diagnose cognitive impairment in patients with PD^7,8,15^. Comparing the classifications obtained with CoMDA with those of the other three screening tests, we objectivated a significant greater reliability of the first one. This result is really worth of attention, as misleading cognitive data could hinder or delay the right diagnosis in patients suffering from PD or APS. Reaching a good classification of cognitive profile is fundamental not only for diagnostic concerns, but also for several clinical and management-related aspects: (i) to select the best pharmacological treatments^36^, (ii) to direct the neurorehabilitation approaches^2,3^, and (iii) to optimize the home-based patient’s management^37^.

The present study highlights how combining psychometric tests and AI can be effective for defining the cognitive profile in PD and APS and for identifying patients at risk of dementia.

The application of predictive analytics through CoMDA-ML for 3-level cognitive-profile classification allowed to reach a reliable level to distinguish among NC, MCI, and IC.

Notably, the crucial condition to reach these two aims is the application of the AI methods. The use of ML in healthcare has become common to carry out regression, classification, or unsupervised clustering tasks for various predictive models. ML implementation is based on loss-function minimization methods, which allow to reduce the expected error in prediction. Notably, the finalized 6-predictor set (i.e., CoMDA, age, education, disease duration, age*CoMDA and education*CoMDA) was not directly anticipated or given by specific hypotheses. The deep-learning process promoted the exclusion of 3 candidate predictors (“gender”, “years of education”, and “L1 score”) and the setting of two additional ones. These last have been obtained by running ML experimentations, with feature-engineering processes, based on the available information, which were the baseline predictors. Interestingly, the two new-built features, used to train the QDA, improved the predictive power, but only together with the other original features.

The present study presents such limitations. Despite the studied population came from all over Italy, this is a single-center study, so that the generalizability of the results is reduced. Further, it could be very interesting to build the same tool by adopting different psychometrics batteries.

Since the rising number of people suffering from PD and APS represents a critical challenge for the public health worldwide, the effort of engaging applicative AI research in this field is really actual. Diagnostic approaches like CoMDA, designed to be equally time-saving and markedly reliable, could maximize our diagnostic capacity and the disease management, thus representing one of the most important steps to follow in the upcoming future.

## Methods

This study is part of a larger prospective, observational, analytical, single-center, cohort study, devised to create a database of clinical, functional, motor, neuropsychological and neurophysiological variables in patients affected by PD and APS coming from all over Italy. The whole study aims at optimally characterizing the profile of these patients in order to better define their treatment into the neurorehabilitative setting.

The protocol was conducted at the Department of Parkinson’s Disease and Movement Disorders Rehabilitation of the “Moriggia-Pelascini” Hospital (Gravedona ed Uniti, Italy) between January 2017 and December 2019. The study design and protocol were approved by the local Ethics Committee (“Comitato Etico Interaziendale delle Province di Lecco, Como e Sondrio”) and were in accordance with the Code of Ethics of the World Medical Association (Declaration of Helsinki, 1967). The study was also registered on ClinicalTrials.gov (NCT04858893).

### Subjects

Six-hundred sixty-one patients were consecutively enrolled by neurologists with experience in movement disorders.

Patients were included in the present study if they met one of the following criteria: (a) diagnosis of idiopathic PD according to the MDS clinical diagnostic criteria^38^; (b) diagnosis of PSP according to the MDS clinical diagnostic criteria^39^; (c) diagnosis of MSA according to the second diagnostic consensus statement^40^ and (d) diagnosis of VP according to Zijlmans et al.^41^.

Exclusion criteria were (a) any focal brain lesion detected with brain-imaging studies; (b) psychiatric disorders, psychosis (evaluated with Neuropsychiatric Inventory), and/or delirium; (c) previous diagnosis of dementia; (d) neurological diseases other than PD or APS; (e) other medical conditions negatively affecting the cognitive status; (f) disturbing resting and/or action tremor, corresponding to scores 2–4 in the specific items of MDS Unified Parkinson’s Disease Rating Scale (MDS-UPDRS) III, such as to affect the psychometric evaluation; (g) disturbing dyskinesia, corresponding to scores 2–4 in the specific items of MDS-UPDRS IV, such as to affect the psychometric evaluation; (h) auditory and/or visual dysfunctions impairing the patient’s ability to perform cognitive tests.

Accordingly, 161 patients were excluded from the study: 76 had previous diagnosis of dementia, 13 presented sensorial deficits (2 with visual impairment, 11 with hearing impairment), 51 suffered from psychiatric disorders and 21 presented disturbing tremor and/or dyskinesia. This led to a final study population of 500 patients. According to the clinical diagnostic criteria^38–41^, patients were classified as follows: 400 with PD, 41 with VP, 31 with PSP, and 28 with MSA.

A complete explanation of the study protocol was provided, and written informed consent was obtained from all participants before their participation in the study.

### Neuropsychological evaluation

The in-depth neuropsychological evaluation was administered by expert neuropsychologists, blinded to patients’ diagnosis. All patients were tested during the morning, in two consecutive days, in a laboratory setting, with constant artificial lighting condition and in the absence of auditory interferences. PD patients were evaluated in medication “on” state.

1. First evaluation: CoMDA
  “CoMDA” stands for “Cognition in Movement Disorders Assessment” and combines MMSE, MoCA and FAB individual measures into a single tool. More specifically, CoMDA consists of all items of the three tests, without repetition for items that appear in more than one of them (e.g., this occurs for the 6 items evaluating orientation, which are both in MMSE and MoCA). CoMDA is thought to maximize the diagnostic-capacity power to screen patients with PD and APS. In our assumption, CoMDA was adopted to define and categorize these patients into three classes: NC, MCI, and IC.
  CoMDA scores result by linear non-weighted combination (additive model) of the non-redundant MMSE, MoCA and FAB items (see Table 1).
  CoMDA allows four different scores in L1: the first three ones are “partial” scores, which are obtained by scoring and summarizing all items of each single test (MMSE: 0–30, MoCA: 0–30, and FAB: 0–18) adjusted (weighted) on the Italian population data as by previous research^42–44^. The fourth one is the “total” score (CoMDA score), which is obtained by summarizing the first three “partial” scores. CoMDA score ranges from 0 (worst performance) to 78 (best performance).
2. Psychometric test battery

Furthermore, patients underwent a large battery of neuropsychological tests for evaluating several cognitive domains (see Table 4), according to the indications provided by Goldman et al.^15^. The majority of studies for obtaining normative values conducted on the Italian population^45–50^ adopted a statistical procedure, which provides regression-based norms and a system of scores on an ordinal scale, named Equivalent Scores (ES). It ranges from class 0 (scores equal or higher than the outer tolerance limit of 5%) to class 4 (scores lower than the median value of the whole sample); 1, 2, and 3 classes were obtained by dividing into three equal parts the area of distribution between 0 and 4. This method makes it possible to judge the scores obtained by the person under examination with respect to those of normal subjects, taking into account of the influence of variables related to age, education, and gender.

**Table 4:** Level 2 neuropsychological evaluation (see the text).

Explored cognitive domain	Administered tests
Executive/frontal functions	1. Wisconsing Card Sorting Test (WCST)
	2. Trail Making Test A & B (TMT A and B)
	3. Stroop Test Error Number (ST-E) and Time (ST-T)
	4. Phonemic fluency (PF)
Visuo-spatial abilities	1. Rey-Osterrieth Complex Figure Test copy (ROCF-C)
	2. Rey-Osterrieth Complex Figure Delayed Recall (ROCF-DR)
Memory	1. Rey-Auditory Verbal Learning Test (RAVLT)
	2. Rey-Auditory Verbal Delayed Recall Test (RAVLT)
	3. Rey-Osterrieth Complex Figure Delayed Recall (ROCF-DR)
Language	1. Categorical Fluency (CF)
	2. Phonemic Fluency (PF)

Hence, the whole patient’s performance was classified based on the termed L2, by adapting the indications provided by Litvan et al.^8^ to the described system of scores, along three consecutive classes: 2 = NC (all ES > 0 or one ES = 0); 1 = MCI (two ES = 0, in tests evaluating the same cognitive domain or two ES = 0, in tests evaluating two different cognitive domains); 0 = IC (more than two ES = 0).

### Baseline statistics and machine learning

#### Descriptive statistics and method-comparison substudy

Basic descriptive statistics for continuous variables were reported as mean ± SD. Descriptive statistics for categorical variables were reported as N (percent frequency).

To assess whether MMSE, MoCA, and FAB scores, derived from CoMDA values, fit the scores computed in the standard way, we set up a method-comparison substudy. A group of 20 patients underwent two assessment sessions, in random order: one session included the administration of MMSE, MoCA, and FAB; the other one included the administration of the CoMDA. The agreement among “standard MMSE, MoCA and FAB scores” and “CoMDA-derived MMSE, MoCA and FAB scores” was assessed by Bland–Altman analysis, computing the bias (systematic difference) and the 95% limits of agreement (the range within which 95% of the differences are expected to lie). The Pearson correlation coefficient was also computed.

The time needed to administer CoMDA was also registered and compared both to the time needed for each standard test and the sum of the times required for the three single tests.

#### Inferential statistics

Non parametric Kruskal–Wallis test and the Chi-square test were carried out for between-group comparisons for continuous and categorical variables respectively. Non parametric Mann–Whitney U-test was applied for single pairwise between-group comparisons. Post hoc comparisons with Dunn–Sidak adjustments were applied for paired multiple-comparison tests.

#### Predictive discrimination analysis

The area under the curve (AUC) of receiver operating characteristic (ROC) curves was computed to assess the ability of all available cognitive screening tools to discriminate between two classes: NC versus MCI or IC, as assessed by the L2 classification (L2 = 0 vs. L2 = 1 + L2 = 2). A value of 0.5 indicates no predictive discrimination, while a value of 1 indicates perfect separation of patients with and without cognitive impairment. The AUC for the CoMDA, MMSE, MoCA, and FAB tools was compared by the Hanley–McNeil test.

A *p*-value < 0.05 was considered statistically significant. All analyses were carried out using the SAS/STAT statistical package, release 9.4 (SAS Institute Inc., Cary, NC, U.S.A.).

#### Machine learning

The ML solutions were engineered to finalize L2 classification on the total sample of 500 patients. All ML models availed of a baseline pool of 7 prescreening candidate predictors: “CoMDA score”, “gender”, “age”, “disease”, “disease duration”, “years of education”, and “L1 score”. A large set of ML architectures (i.e., algorithms, parameters and hyperparameter combinations) were concurrently tested to obtain the final model, which is the best in fitting algorithmic configuration to correctly predict L2 classification from the available data.

Models were validated via k-fold cross-validation operated on a training partition set out of the 500 available samples. This procedure implies to split the available dataset into k non-overlapping folds. Each of the k folds could be used as a held-back test set, while all other folds collectively are used as a training dataset. A test/holdout set was used to measure unbiased cross-generalization performance level of the ML solution on unseen data. A random-shuffling train-test split was carried out to avoid any potential selection bias. This procedure was performed on the original data sample before any model-training operation.

To know the most important predictor among the 7 available, the value of Information Gain, obtained in prediction, has been quantified. This value reflects a measure of “entropy reduction” or “information relevance” of predictors in the dataset of reference^51,52^.

Finally, cross-algorithm performances were assessed by widely adopted standard prediction metrics: accuracy, AUC, recall, precision, F1, kappa and MCC.

All machine-learning experiments were carried out by coding in Python 3.8 (Python Software Foundation, 9450 SW Gemini Dr., ECM# 90772, Beaverton, OR 97008, USA) with full use of PyCaret 2.3.3 library (PyCaret.org. PyCaret) and Jupiter Notebook (Python editors). The finalized version of ML algorithm had hyperparameters fine-tuned via “Optuna” mathematical method^53^.

## Supplementary information

Supplementary tables

## Data Availability

The data that support the findings of this study are available from the corresponding author upon reasonable request.

## Code availability

All source codes and models are available for consultation by the authors’ permission.
