# Supplementary material for: Optimization of cognitive assessment in Parkinsonisms by applying artificial intelligence to a comprehensive screening test
Source: NPJ Parkinsons Dis. 2022 Apr 11;8:42. doi: 10.1038/s41531-022-00304-z (PMC9001753; doi:10.1038/s41531-022-00304-z)
Supplement: Supplementary file 1 — Supplementary tables [file 41531_2022_304_MOESM1_ESM.docx]

**Supplementary tables**

**e-table 1.** *Agreement between MMSE, MOCA and FAB scores and the same scores obtained from CoMDA - Bland-Altman analysis (see the text).*

| **Variable** | CoMDA based | True | Bias | 95%LoA | Pearson r |
| --- | --- | --- | --- | --- | --- |
| MMSE  (total score) | 28.4±1.4 | 28.4±1.6 | 0.1 | -1.8÷1.9 | 0.82 |
| MMSE  (correct score) | 28.4±1.6 | 28.3±1.9 | 0.2 | -1.5÷1.8 | 0.90 |
| MoCA  (total score) | 24.3±2.5 | 24.0±2.7 | 0.3 | -1.2÷1.8 | 0.96 |
| MoCA  (correct score) | 24.6±2.5 | 24.3±2.7 | 0.3 | -1.2÷1.8 | 0.96 |
| FAB  (total score) | 16.1±1.4 | 15.9±1.7 | 0.1 | -1.3÷1.6 | 0.90 |
| FAB  (correct score) | 16.1±1.5 | 15.9±1.6 | 0.3 | -1.4÷1.9 | 0.85 |

**Abbreviations***: MMSE (Mini-Mental State Examination); FAB (Frontal Assessment Battery); MoCA (Montreal Cognitive Assessment).*

**e-table 2**. *Neuropsychological mean scores for all patients and percentages of normal performances for each specific disease-group.*

| **Variable** | **All patients**  **(mean scores)** | **PD*** | **MSA*** | **PSP*** | **VP*** |
| --- | --- | --- | --- | --- | --- |
| WCST | 74.40±35.86 | 57.25 | 82.1 | 38.7 | 43.9 |
| ST-E | 5.89±7.89 | 60 | 64.3 | 19.4 | 24.4 |
| ST-T | 21.55±14.87 | 86.5 | 75 | 64.5 | 73.2 |
| TMT-A | 49.52±53.10 | 88.25 | 71.4 | 58.1 | 75.6 |
| TMT-B | 134.10±102.61 | 80.25 | 75 | 51.6 | 51.2 |
| TMT B-A | 94.56±83.23 | 79.25 | 71.4 | 45.2 | 48.8 |
| RAVLT-e | 42.77±10.00 | 89 | 89.3 | 87.1 | 90.2 |
| RAVLT-dr | 8.63±3.17 | 87.5 | 82.1 | 77.4 | 90.2 |
| ROCF-c | 28.35±7.24 | 59.75 | 32.1 | 16.1 | 48.8 |
| ROCF-dr | 14.66±5.77 | 79.5 | 67.9 | 48.4 | 73.1 |
| CF | 42.05±9.88 | 88.5 | 78.6 | 64.5 | 85.4 |
| PF | 30.87±10.82 | 86.25 | 75 | 51.6 | 82.9 |

**Abbreviations:** *PD (Parkinson’s disease); MSA (Multi System Atrophy); PSP (Progressive Supranuclear Palsy); VP (Vascular Parkinsonism).; MMSE (Mini-Mental State Examination); FAB (Frontal Assessment Battery); MoCA (Montreal Cognitive Assessment); CoMDA (Cognitive Screening in Movement Disorders Assessment); WCST (Wisconsin Card Sorting Test); TMT (Trial Making Test); ST –E and –T (Stroop Test – Errors and Time); RAVLT -e and –dr (Ray Auditory Verbal Learning Test – encoding and delayed recall); ROCF –c and dr (Rey–Osterrieth Complex Figure Test – encoding and delayed recall), CF (Categorical Fluency), PF (Phonemic Fluency). *= percentage of normal performances.*

**e-table 3.** *Cross tabulation of L1 cognitive impairment by patients’ groups. Percentage is expressed in brackets (see the text).*

| **Variable** | PD | MSA | PSP | VP | Total |
| --- | --- | --- | --- | --- | --- |
| MMSE | 32  (8) | 4  (1,8) | 5  (2,2) | 3  (1,3) | 44  (9) |
| MoCA | 9  (2,3) | 1  (3,6) | 4  (21,9) | 3  (7,3) | 17  (3,4) |
| FAB | 133  (33) | 10  (35,7) | 18  (58) | 19  (46,3) | 180  (36) |
| Total | 400 | 28 | 31 | 41 | 500 |

**Abbreviations:** *PD (Parkinson’s disease); MSA (Multi System Atrophy); PSP (Progressive Supranuclear Palsy); VP (Vascular Parkinsonism).*

**e-table 4.** *Level 2 cognitive profile cross-tabulation by patients’ groups. Percentage is expressed in brackets (see the text).*

| **Variable** | PD | MSA | PSP | VP | Total |
| --- | --- | --- | --- | --- | --- |
| L2=0 | 113  (28,3) | 12  (42,9) | 24  (77,4) | 18  (43,9) | 167  (33,4) |
| L2=1 | 101  (25,3) | 5  (17,9) | 3  (9,7) | 17  (41,5) | 127  (25,4) |
| L2=2 | 186  (46,4) | 11  (39,2) | 4  (12,9) | 6  (14,6) | 206  (41,2) |
| Total | 400 | 28 | 31 | 41 | 500 |

*Chi-Square p<0.0001*

**Abbreviations:** *PD (Parkinson’s disease); MSA (Multi System Atrophy); PSP (Progressive Supranuclear Palsy); VP (Vascular Parkinsonism); L2 (Level 2).*

**e-table 5.** *Comparison values of MMSE scores, MoCA scores, FAB scores and CoMDA scores in IC versus MCI and IC or cognitive impairment (see the text).*

| **Variable** | NC  (L2=2) | MCI+IC  (L2=1+0) | p-value NC vs MCI+IC |
| --- | --- | --- | --- |
| MMSE scores | 28.06±1.82 | 26.35±2.59 | <0.0001 |
| MoCA scores | 25.17±2.61 | 21.54±3.75 | <0.0001 |
| FAB scores | 15.61±2.13 | 13.13±2.90 | <0.0001 |
| CoMDA scores | 68.84±4.66 | 61.02±7.80 | <0.0001 |

**Abbreviations***: MMSE (Mini-Mental State Examination); FAB (Frontal Assessment Battery); MoCA (Montreal Cognitive Assessment); COMDA (Cognition in Movement Disorders Assessment).*

**e-table 6.** *Averaged metric performances of 15 baseline algorithms comparison, after training set.*

| **Model** |  | **Accuracy** | **AUC** | **Recall** | **Prec.** | **F1** | **Kappa** | **MCC** | **TT (Sec)** |
| --- | --- | --- | --- | --- | --- | --- | --- | --- | --- |
| **nb** | Naive Bayes | 0.6102 | 0.7738 | 0.5603 | 0.6064 | 0.5907 | 0.3878 | 0.4010 | 0.0060 |
| **qda** | **Quadratic Discriminant Analysis** | **0.6071** | **0.7647** | **0.5695** | **0.6212** | **0.5976** | **0.3882** | **0.3979** | **0.0070** |
| **lr** | Logistic Regression | 0.6018 | 0.7861 | 0.5305 | 0.5171 | 0.5373 | 0.3596 | 0.3878 | 0.5180 |
| **ridge** | Ridge Classifier | 0.5961 | 0.0000 | 0.5194 | 0.4620 | 0.5138 | 0.3440 | 0.3801 | 0.0050 |
| **lda** | Linear Discriminant Analysis | 0.5960 | 0.7731 | 0.5277 | 0.5404 | 0.5422 | 0.3524 | 0.3762 | 0.0060 |
| **et** | Extra Trees Classifier | 0.5816 | 0.7465 | 0.5346 | 0.5507 | 0.5592 | 0.3449 | 0.3511 | 0.0780 |
| **rf** | Random Forest Classifier | 0.5702 | 0.7544 | 0.5140 | 0.5384 | 0.5400 | 0.3243 | 0.3352 | 0.0900 |
| **ada** | Ada Boost Classifier | 0.5646 | 0.7074 | 0.5145 | 0.5395 | 0.5454 | 0.3211 | 0.3268 | 0.0380 |
| **catboost** | CatBoost Classifier | 0.5560 | 0.7435 | 0.5119 | 0.5364 | 0.5393 | 0.3101 | 0.3150 | 1.1180 |
| **dt** | Decision Tree Classifier | 0.5304 | 0.6414 | 0.4993 | 0.5343 | 0.5249 | 0.2746 | 0.2782 | 0.0060 |
| **lightgbm** | Light Gradient Boosting Machine | 0.5303 | 0.7268 | 0.4872 | 0.5163 | 0.5174 | 0.2723 | 0.2759 | 0.0730 |
| **gbc** | Gradient Boosting Classifier | 0.5302 | 0.7299 | 0.4831 | 0.5099 | 0.5132 | 0.2691 | 0.2741 | 0.1090 |
| **xgboost** | Extreme Gradient Boosting | 0.5245 | 0.7147 | 0.4777 | 0.5037 | 0.5071 | 0.2610 | 0.2660 | 0.3100 |
| **knn** | K Neighbors Classifier | 0.4154 | 0.5992 | 0.3940 | 0.4223 | 0.4096 | 0.1000 | 0.1015 | 0.0110 |
| **svm** | SVM - Linear Kernel | 0.3610 | 0.0000 | 0.3509 | 0.2116 | 0.2363 | 0.0303 | 0.0429 | 0.0080 |

**e-table 7.** Information Gain for CoMDA score in Original Dataset.

| **Original Dataset** |
| --- |
| Use of Information Gain (IG) algorithm |
| **Features Information Gain*** |
| CoMDA score 0.150350 |
| Age 0.052968 |
| Education 0.025740 |
| Disease 0.014435 |

********Information Gain algorithm is IG(S, a) = H(S) – H(S | a), where IG(S, a) is the information for the dataset S for the variable a for a random variable, H(S) is the entropy for the dataset before any change (described above) and H(S | a) is the conditional entropy for the dataset given the variable a (Source: Information Gain and Mutual Information for Machine Learning - Jason Brownlee on October 16, 2019 -* [*https://machinelearningmastery.com/information-gain-and-mutual-information/*](https://machinelearningmastery.com/information-gain-and-mutual-information/)*).*

**e-table 8**. Information Gain for CoMDA score in training set for Quadratic Discriminant Analysis.

| **Train set used for Learning Quadratic Discriminant Analysis** |
| --- |
| Use of Information Gain (IG) algorithm |
| **Features Information Gain*** |
| CoMDA score 0.171217 |
| Education*CoMDA score 0.164859 |
| Age 0.085967 |
| Education 0.024135 |
| Disease 0.013458 |
| Age*CoMDA score 0.000000 |

********Information Gain algorithm is IG(S, a) = H(S) – H(S | a), where IG(S, a) is the information for the dataset S for the variable a for a random variable, H(S) is the entropy for the dataset before any change (described above) and H(S | a) is the conditional entropy for the dataset given the variable a (Source: Information Gain and Mutual Information for Machine Learning - Jason Brownlee on October 16, 2019 -* [*https://machinelearningmastery.com/information-gain-and-mutual-information/*](https://machinelearningmastery.com/information-gain-and-mutual-information/)*).*

**e-table 9.** *Hyper-parameter modelling and optimization.*

| **Model internals at cross-validation (training set)** |
| --- |
| *Trained model (k-fold cross generalization) before hyper-parameters optimization* |
| **Default Hyper-parameters** |
| Priors* None |
| reg_param** 0.0 |
| store_covariance*** False |
| Tolerance**** 0.0001 |
|  |
| **Finalized Model internals (test set)** |
| *Finalised and tuned model with optimized hyper-parameters* |
| **Tuned Hyper-parameters** |
| priors None |
| reg_param 0.484858 |
| store_covariance False |
| tolerance 0.0001 |

**(class proportions are inferred from the training data)*

***(Regularizes the per-class covariance estimates by transforming S2 as S2 = (1 - reg_param) * S2 + reg_param * np.eye(n_features), where S2 corresponds to the scaling_ attribute of a given class.)*

****(If True, the class covariance matrices are explicitly computed and stored in the self.covariance_ attribute.)*

*****(Absolute threshold for a singular value to be considered significant, used to estimate the rank of Xk where Xk is the centered matrix of samples in class k. This parameter does not affect the predictions. It only controls a warning that is raised when features are considered to be collinear)*

**e-table 10.** *CoMDA model: prediction performance on 10 random samples.*

|  | **Age** | **Disease** | **Edu** | **CoMDA score** | **CoMDA score*Age** | **CoMDA score*Edu** | **L2** | **Label** | **Score** |
| --- | --- | --- | --- | --- | --- | --- | --- | --- | --- |
| **0** | 78.0 | 11.0 | 13.0 | 58.639999 | 4573.919922 | 762.320007 | MCI | IC | 0.5902 |
| **1** | 77.0 | 6.0 | 6.0 | 55.419998 | 4267.339844 | 332.519989 | IC | IC | 0.8246 |
| **2** | 80.0 | 11.0 | 5.0 | 57.160000 | 4572.799805 | 285.799988 | IC | IC | 0.8835 |
| **3** | 82.0 | 3.0 | 8.0 | 62.060001 | 5088.919922 | 496.480011 | IC | MCI | 0.8751 |
| **4** | 78.0 | 16.0 | 5.0 | 54.930000 | 4284.540039 | 274.649994 | IC | IC | 0.9766 |
| **5** | 59.0 | 14.0 | 8.0 | 73.750000 | 4351.250000 | 590.000000 | NC | NC | 0.9326 |
| **6** | 64.0 | 14.0 | 5.0 | 71.580002 | 4581.120117 | 357.900024 | NC | NC | 0.7858 |
| **7** | 71.0 | 4.0 | 8.0 | 63.619999 | 4517.020020 | 508.959991 | IC | MCI | 0.5453 |
| **8** | 68.0 | 5.0 | 5.0 | 66.050003 | 4491.400391 | 330.250000 | IC | MCI | 0.5044 |
| **9** | 55.0 | 5.0 | 8.0 | 72.720001 | 3999.600098 | 581.760010 | NC | NC | 0.9828 |
